# Intercostal artery’s access for type II endoleak embolization

**DOI:** 10.1093/icvts/ivad063

**Published:** 2023-05-04

**Authors:** Veronica Lorenz, Luigi Muzzi, Laura Candeloro, Carmelo Ricci, Marco Cini, Giuseppe Alba, Enrico Tucci, Eugenio Neri

**Affiliations:** Aortic Surgery Unit, Siena University Hospital, Siena, Italy; Aortic Surgery Unit, Siena University Hospital, Siena, Italy; Interventional Radiology Unit, Siena University Hospital, Siena, Italy; Interventional Radiology Unit, Siena University Hospital, Siena, Italy; Interventional Radiology Unit, Siena University Hospital, Siena, Italy; Aortic Surgery Unit, Siena University Hospital, Siena, Italy; Aortic Surgery Unit, Siena University Hospital, Siena, Italy; Aortic Surgery Unit, Siena University Hospital, Siena, Italy

**Keywords:** Endoleak, Intercostal artery, Coil embolization, Thoracic endovascular aneurysm repair, Endovascular aneurysm repair

## Abstract

Endoleaks represent a main issue of endovascular approach of thoracic aorta diseases and their treatment continue to be challenging. According to some authors, type II endoleaks sustained by intercostal arteries should not be treated because of the technical difficulties. However, the persistence of a pressurized aneurysmal may confer an ongoing risk of enlargement and/or aortic rupture. We describe the successful treatment of type II endoleak in 2 patients with an intercostal artery’s access. In both cases, the endoleak was discovered during follow-up and was treated with its direct coil embolization under local anaesthesia.

## INTRODUCTION

Endovascular repair improved the results of thoracic and abdominal aortic aneurysm treatment [1]. However, one of the most common complications after endovascular aneurysm repair (EVAR) or thoracic EVAR is represented by endoleaks [2]. The best strategy for treating type II endoleaks is still debated [3], and, according to some authors, they should be treated conservatively [4, 5].

We want to report our experience in the successful intercostal artery (IA) coil embolization in 2 patients with type II endoleak.

The consent to publish this report was given by both patients.

## FIRST PATIENT

**Figure 1:**
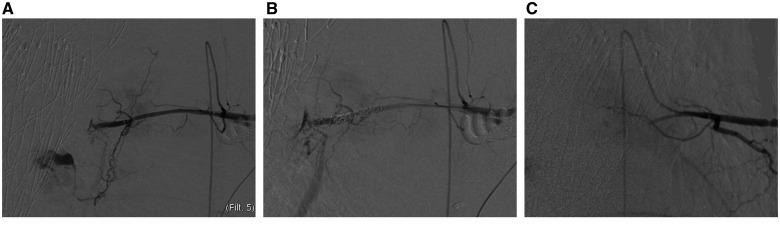
(**A**) Evidence of Adamkiewicz artery. (**B**) Intercostal artery embolization with 2 controlled-release coils (Azur-Cx) upstream and downstream of the origin of the collateral. (**C**) Final result.

A 65-year-old female, a heavy smoker with hypertension and chronic obstructive pulmonary disease. She was admitted to our department with the diagnosis of ‘mega aorta’ extending from the sinotubular junction to the thoraco-abdominal aorta where it measured 7 cm in maximum diameter (Fig. 1). The aneurysm involved the origin of both superior mesenteric artery and the coeliac trunk ending just above the origin of renal arteries. Eccentric parietal thrombosis was present in the distal part of the descending thoracic aorta (Video 1).

Based on the anatomic features, we decided to perform a two-staged hybrid repair consisting in aortic arch replacement, using the elephant trunk technique with the soft Gelweave Siena Graft prosthesis (Terumo Aortic; Inchinnan, Glasgow, UK) and endovascular completion 25 days after first-stage repair.

The patient underwent stent grafting of the thoraco-abdominal aneurysm with 2 stent grafts type Gore C-tag (W.L. Gore and Associates, Flagstaff, AZ), preserving the perfusion of the coeliac trunk and superior mesenteric artery using the chimney grafts technique with two 8 mm × 150 mm GORE VIABAHN (W.L. GORE, Flagstaff, AZ, USA) endografts.

However, 1-month follow-up computed tomography (CT) angiography showed a type II endoleak sustained by the 9th and 10th right intercostal arteries. Our decision was not to treat immediately the endoleak and to wait for a possible spontaneous closure of the intercostal arteries.

A CT scan performed 6 months later, demonstrated the spontaneous closure of the 9th IA; nevertheless, the 10th IA still appeared to perfuse the aneurysmal sac; the aneurysm also showed a slight increase in dimensions at this time (1.3 cm wider); therefore we decided to treat the endoleak. After a thorough imaging analysis and precise identification of surgical reference points of the target artery, we decided to perform direct coil embolization.

## SECOND PATIENT

A 76-year-old male with severe peripheral artery disease and chronic renal insufficiency had a history of frequent hospitalization for both respiratory and heart failure.

During the last 20 years, he had undergone surgical resection of the infrarenal abdominal aortic aneurysm with bisiliac prosthesis implantation. After 10 years he underwent the implantation of a branched endoprosthesis (BEVAR) and more recently he underwent the stenting of the superior mesenteric artery due to type III c endoleak with enlargement of the aneurysm sac.

At CT scan follow-up, a type II endoleak supplied from the 9th left IA was demonstrated. Therefore, we decided to treat the endoleak due to progressive sac dilatation (1.6 cm in 9 months).

Given the general conditions, the chronic renal insufficiency, the previous abdominal surgery and particularly the severe peripheral atherosclerosis, we decided to proceed with a less-invasive intervention and treat the endoleak with an endovascular procedure with intercostal access.

## INTERVENTION

For both patients, in the prone position, the correct intercostal space was identified. Under local anaesthesia, the IA was exposed through a limited para-vertebral incision (4–5 cm) and the section of the external and internal intercostal muscles. Care was taken to avoid the opening of the pleural space. After systemic heparinization, by means of a coaxial type Terumo Progreat microcatheter with 0.018″ nitinol guidewire (Progreat, Terumo, Tokyo, Japan) was advanced in the aneurysmal sac using the puncture needle to introduce the wire.

After a diagnostic angiogram that confirmed the correct positioning in the target vessel, we performed a coil embolization by 2 controlled-release coils (Azur-Cx 3 mm × 8 cm, Terumo, Tokyo, Japan) upstream and downstream of the origin of the aorta collateral (Fig. 1).

Final angiographic control demonstrated a satisfactory result of the procedure with no signs of residual endoleak. At the end of the procedure, the guidewire was removed and IA was ligated with vascular clip. The patient was discharged after 2 days.

The CT images follow-up at 3 months showed the excellent result of the procedure for both patients, with the perfect exclusion of the endoleak without other complications.

## DISCUSSION

Endovascular repair is widely used to treat extensive thoracic and abdominal aneurysmal lesions of the aorta.

Although less-invasive, endovascular solutions are burdened by the risk of complication, including endoleaks. It is estimated that the incidence of endoleak after thoracic endovascular aneurysm repair is similar to EVAR and is about 5–10% of the cases [6]. Their management continues to be a challenge in the endovascular approach to both abdominal and thoracic aorta diseases, conferring a need for long-term surveillance or reintervention [7]. Whatever the cause, endoleaks may continue to perfuse and pressurize the aorta, thereby conferring an ongoing risk of aneurysm enlargement and/or aortic rupture.

Nevertheless, in literature is frequently reported that type II endoleak, sustained by IA, is usually left untreated because of technical difficulties and serious risk of possible spinal cord ischaemia when attempting their treatment [8]. Endoleak from IA, according to some authors [6, 9], should not be treated at all.

Trans arterial or direct puncture embolization of type II thoracic endoleaks can be considerably complex because collateral circulation in the thoracic aorta is less developed than in the abdominal tract. In addition, differently from the translumbar embolization of abdominal endoleaks, a direct thoracic puncture to access thoracic endoleaks may expose to high procedural complications.

Nonetheless, as sac may continue to expand, it is uncertain whether the advocated watchful waiting approach to type II thoracic endoleak is warranted.

Ronald *et al.* [10] recently described the direct percutaneous IA access to treat thoracic type II endoleaks. However, in our opinion, this procedure is not feasible in obese patients and exposes to the risk of artery perforation and complication such as local haematoma. A small surgical incision allows a better view and control of the artery and is, in our opinion, safer, as also described by Karube *et al.* [11]. The authors reported a single surgical exposure of intercostal arteries to perform embolization of feeding arteries directly. Our experience with 2 patients confirms the feasibility of the procedure.

## CONCLUSIONS

In our experience, embolization of endoleak type II, through IA, resulted in a technically feasible procedure, requiring low doses of contrast, without problems even for the patient with renal insufficiency. Collaboration between cardiovascular surgeons and interventional radiologists is the key to success.

**Conflict of interest:** None declared.

### Reviewer information

Interdisciplinary CardioVascular and Thoracic Surgery thanks Roman Gottardi, Victor X. Mosquera and the other, anonymous reviewer(s) for their contribution to the peer review process of this article.

## Data Availability

Data available on request.
